# Phosphatidylinositol 3-Kinase γ Is Required for the Development of Experimental Cerebral Malaria

**DOI:** 10.1371/journal.pone.0119633

**Published:** 2015-03-16

**Authors:** Norinne Lacerda-Queiroz, Fatima Brant, David Henrique Rodrigues, Juliana Priscila Vago, Milene Alvarenga Rachid, Lirlândia Pires Sousa, Mauro Martins Teixeira, Antonio Lucio Teixeira

**Affiliations:** 1 Laboratory of Immunopharmacology/Department of Biochemistry and Immunology, Institute of Biological Sciences, Federal University of Minas Gerais, Belo Horizonte, Brazil; 2 Department of Clinical and Toxicological Analysis, Faculty of Pharmacy, Federal University of Minas Gerais, Belo Horizonte, Brazil; 3 Interdisciplinary Laboratory of Medical Investigation, School of Medicine, Federal University of Minas Gerais, Minas Gerais, Brazil; Agency for Science, Technology and Research—Singapore Immunology Network, SINGAPORE

## Abstract

Experimental cerebral malaria (ECM) is characterized by a strong immune response, with leukocyte recruitment, blood-brain barrier breakdown and hemorrhage in the central nervous system. Phosphatidylinositol 3-kinase γ (PI3Kγ) is central in signaling diverse cellular functions. Using PI3Kγ-deficient mice (PI3Kγ^-/-^) and a specific PI3Kγ inhibitor, we investigated the relevance of PI3Kγ for the outcome and the neuroinflammatory process triggered by *Plasmodium berghei* ANKA (PbA) infection. Infected PI3Kγ^-/-^ mice had greater survival despite similar parasitemia levels in comparison with infected wild type mice. Histopathological analysis demonstrated reduced hemorrhage, leukocyte accumulation and vascular obstruction in the brain of infected PI3Kγ^-/-^ mice. PI3Kγ deficiency also presented lower microglial activation (Iba-1+ reactive microglia) and T cell cytotoxicity (Granzyme B expression) in the brain. Additionally, on day 6 post-infection, CD3^+^CD8^+^ T cells were significantly reduced in the brain of infected PI3Kγ^-/-^ mice when compared to infected wild type mice. Furthermore, expression of CD44 in CD8+ T cell population in the brain tissue and levels of phospho-IkB-α in the whole brain were also markedly lower in infected PI3Kγ^-/-^ mice when compared with infected wild type mice. Finally, AS605240, a specific PI3Kγ inhibitor, significantly delayed lethality in infected wild type mice. In brief, our results indicate a pivotal role for PI3Kγ in the pathogenesis of ECM.

## Introduction

Cerebral malaria (CM) is a severe neurological syndrome in humans, resulting from *Plasmodium falciparum* infection. Despite significant developments in the area, there are several aspects of the pathogenesis of CM that remain incompletely understood [1, 2]. Many animal models have been developed to elucidate the inflammatory and/or immunological mechanisms involved in CM, especially using murine models in which animals are experimentally infected with *Plasmodium berghei* ANKA (PbA) strain [3, 4]. Although animal models do not reproduce human disease exactly, they do exhibit some histopathological similarities including changes in the cerebral microvasculature, breakdown of the blood-brain barrier (BBB), petechial hemorrhages, congestion and edema in the brain [5]. The activation and recruitment of T lymphocytes into the brain, in particular CD8+ T cells, are crucial steps in the development of experimental CM (ECM) [6–8]. Activated CD8+ T lymphocytes that accumulate in the brain have a major role in the onset of the neurological symptoms through a cytotoxic mechanism dependent of Granzyme B and perforin [9, 10]. In addition to central nervous system (CNS) involvement, mice can develop pulmonary disease, with edema, interstitial inflammatory cell accumulation and microhemorrhages (reviewed in [11]).

Phosphatidylinositol-3-kinases (PI3Ks) are a subfamily of lipid kinases that play a key role in intracellular signaling and are involved in several cellular responses, including actin rearrangement and polarization, and leukocyte activation and migration into inflamed tissues [12]. The PI3K subfamily is divided into three classes with different isoforms, PI3Kγ being the unique member of class IB and it is activated by G protein-coupled receptors and mainly expressed in leukocytes [13]. Class IA and Class IB PI3K (including PI3Kγ) subtypes have been implicated in the migration of activated T cells [14, 15]. Accordingly, inhibition of PI3Kγ pathway, by AS605240, has been considered a potential therapeutic strategy to treat various T-lymphocyte-dependent diseases, including autoimmune and inflammatory diseases [13, 15–17]. However, the role of PI3K has not been determined in ECM, a T-cell mediated pathology.

Therefore, the main objective of this work was to investigate the role of PI3Kγ in the outcome of PbA infection and the relevance of this molecule for the associated inflammatory process. We found that the absence of PI3Kγ significantly delayed mortality and reduced clinical signs and histopathological brain changes associated with by PbA infection.

## Material and Methods

### Mice

Wild-type (WT) C57BL/6 (female and 6 to 8-wk-old) mice were obtained from the Animal Care Facilities of Universidade Federal de Minas Gerais (UFMG-Brazil). PI3Kγ^-/-^ mice, with C57BL/6 genetic background, were a kind gift from Dr. Josef M. Penninger and were bred and maintained under specific pathogen free conditions at Instituto de Ciências Biológicas, UFMG (ICB-UFMG). Age- and sex-matched PI3Kγ^-/-^ mice were used. The Animal Ethics Committee of UFMG approved all experimental procedures used (protocol number: 193/06).

### Experimental Malaria Infection

Blood stages of *P*. *berghei* strain ANKA constitutively expressing green fluorescent protein (*P*. *berghei* ANKA-GFP) (15cy1 clone) was used (gift from Dr. Claudio Marinho, University of São Paulo). Mice were infected via intraperitoneal injection of 10^6^ parasitized erythrocytes as previously described [18]. Mice were observed daily, and clinical neurological signs of ECM (ataxia, paralysis, and coma) were assessed and cumulative ECM incidence after infection was reported. Mice presenting neurological symptoms of ECM were anesthetized with ketamine/xylazine (150 mg/kg, 10 mg/kg, respectively; Syntec, Brazil) before cervical dislocation and/or tissue collection. All Kaplan—Meier graphs of mouse survival represent mice that were found dead or reached moribundity and were euthanized.

### Parasitemia

Parasitemia was assessed in 2μL of blood collected from the tail vein after infection with EGFP-PbA. Blood was diluted in 3 ml of PBS containing 0.5% bovine serum albumin, and fluorescent cells were analyzed using a flow cytometer (FACScan, Becton Dickinson, San Jose, CA, USA) and FlowJo software (TreeAge Software, Inc, Williamstown, MA, USA).

### Histological Analysis

Mice were euthanized and perfused with intracardiac sterile PBS/EDTA, 0.002 mmol/L, to remove circulating red blood cells and leukocytes from the circulation. Brain and lungs were immediately removed and fixed in 10% buffered formalin for histological analysis. Longitudinal sections (4μm) were stained with hematoxylin and eosin (H&E), which were evaluated by a pathologist blinded to the experiment. Brain microvascular obstruction was evaluated by scoring the number of vessels containing sequestered blood cells, as described by Fauconnier et al. [19], on multiple fields (n = 30 sections) corresponding to whole brain sections. The area of hemorrhage from cerebellum was measured in 10 sections obtained for each animal using the software Image Pro-Plus and results were expressed as the hemorrhagic foci/field [20]. Sections were captured with a digital camera (Optronics DEI-470) connected to a microscope (Olympus IX70) with a magnification of x200.

Lung pathology was quantified using a semiquantitative score with increasing severity of changes (0 to 5), as assessed by Fauconnier et al. [19]. Lung sections were scored on 10 fields per section for erythrocyte accumulation in alveoli (less than 1 cell per alveoli = 0, 1 cell per alveoli = 1, 2 to 5 cells per alveoli = 2, 5 to 10 cells per alveoli = 3, 10 to 30 cells per alveoli = 4, and 30 cells pre alveoli = 5); alveolar size (all normal alveoli = 0, mix of normal and medium-sized alveoli = 1, medium-sized alveoli only = 2, mix of medium-sized and small alveoli = 3, all small alveoli = 4, and full obstruction = 5); and thickening of alveolar septae due to inflammatory cells and edema was scored with increasing severity from 0 to 5 as an estimate of interstitial inflammation.

### Immunohistochemistry

Microglial activation was analyzed by immunohistochemistry using the antibody to ionized calcium-binding adaptor molecule 1 (Iba-1) from Wako Chemicals USA Inc. (Richmond, VA, USA). Brain sections (4 μm) from formalin-fixed, paraffin-embedded tissue were submitted to antigen retrieval with citrate buffer (pH 6) at 95°C for 20 minutes, washed, and blocked for endogenous peroxidase activity (3% H_2_O_2_ in PBS/bovine serum albumin 1%) for 30 minutes. Slides were then washed and blocked with PBS containing 5% (w/v) nonfat dry milk and 0.1% Tween-20. All incubations were performed in a wet chamber. Brain sections were then incubated overnight at 4°C with rabbit monoclonal antibody, which was diluted 1:300. Universal Dako Biotinylated link (Dako, Carpinteria, CA, USA) were applied and after washing, sections were incubated with peroxidase substrate (diaminobenzidine—DAB) (Dako, Carpinteria, CA, USA) protected from light. Sections without primary antibodies were equally processed to control for unspecific binding. Finally, sections were counterstained with Gill’s hematoxylin, washed and mounted. Images of 10 fields from brain regions per animal were digitized using a ×200 planachromatic objective. Quantification of microglia activation was performed in a blind fashion by a single observer and the result was expressed as number of activated cells per field.

### Lysate preparation and western blot analysis

Western blot analysis for Granzyme B and phospho-IκB-α were performed as described elsewhere [20]. Briefly, whole cell extracts were obtained from homogenized brains by using a lyses buffer (1% (v/v) Triton X-100, 100 mM Tris/HCl, pH 8.0, 10% (v/v) glycerol, 5 mM EDTA, 200 mM NaCl, 1 mM DTT, 1 mM PMSF, 25mM NaF, 2.5 μg/ml leupeptin, 5 μg/ml aprotinin, and 1mM sodium orthovanadate). Lysates were centrifuged at 13,000×*g* for 10min at 4°C and quantified using the Bradford assay reagent from Bio-Rad (Hercules, CA). Protein extracts (40μg) were separated by electrophoresis on a denaturing 10% polyacrylamide-SDS gel and transferred onto nitrocellulose membranes. Membranes were blocked overnight at 4°C with PBS containing 5% (w/v) non-fat dry milk and 0.1% Tween-20 and washed three times with PBS containing 0.1% Tween-20. The membranes were then incubated in primary antibody anti-phospho-IκB or anti-Granzyme B (Cell Signaling Technology—Beverly, MA, USA) diluted in phosphate-buffered saline containing 5% (w/v) BSA and 0.1% Tween-20. After washing, membrane was incubated with horseradish peroxidase-conjugated secondary antibody. Immunoreactive bands were visualized by using an enhanced chemiluminescence detection system, as described by the manufacturer (GE Healthcare, Piscataway, NJ, USA). The protein levels were quantified using ImageJ software (NIH, Bethesda, MD), and the values were normalized to the values of β-actin. The results were expressed as phospho-IκB-α/ β-actin or Granzyme B/ β-actin ratio, measured in arbitrary units (AU).

### Isolation of leukocytes in the brain

Mice were euthanized on day 6 p.i., a time point when all WT mice showed neurological symptoms of ECM. They were perfused intracardially with PBS to remove both circulating and non-adherent RBCs and leukocytes. Brains were removed and adherent leukocytes isolated using previously described protocols [19, 21, 22]. For brain analysis, each sample comprised a pool of 2 mice brains (n = 4 samples per group). Briefly, brains were collected and homogenized kindly using the sterile glass tissue grinder in RPMI 1640 medium containing 5% FCS. Brain homogenates were passed through a nylon cell strainer (70 μm; Becton Dickinson, Brazil) and then centrifuged at 400×*g* for 10 minutes. The pellet was resuspended in 35% Percoll gradient (Sigma-Aldrich, St. Louis, MO) and this was deposited on a 70% Percoll gradient. After centrifugation (1,100×g), leukocytes were collected at the boundary layer, between the 70% and 35% gradient [22]. Afterwards, leukocytes were resuspended in FACS buffer (PBS containing 1% BSA and 0.01% NaN_3_) and counted.

### Immunolabeling and flow cytometric analysis

Brain leukocytes were stained for extracellular molecular expression patterns using fluorescence labeled antibodies for 30 minutes: mouse CD3 (BD Pharmingen San Diego, CA; clone 17A2), CD8α (from BD Pharmingen San Diego, CA; clone 53–6.7), CD44 (eBioscience San Diego, CA; clone IM-7), and isotype controls (all from Pharmingen San Diego, CA). Intracellular staining for Granzyme B (Invitrogen Grand Island, NY; clone GB11) was performed after cell fixation. The cells were then phenotyped by flow cytometry and, for each sample, 30,000–50,000 cells were scored. The frequency of positive cells was analyzed using gates that took into account size (forward light scatter) and granularity (side light scatter) for lymphocytes. Limits for the quadrant markers were always set based on negative populations and isotype controls. Data were acquired on a FACScan (Becton Dickinson, San José, CA, USA) and analysed using the FlowJo 7.5.3 software (TreeStar Inc., Ashland, OR, USA). Results are presented as absolute number of cells per brain, percentage or mean fluorescence intensity (MFI).

### IFN-γ and CCL5 measurement in the serum

The levels of IFN-γ and CCL5 were quantified in serum of control and PbA-infected mice on day 6 p.i., using DuoSet ELISA kits (R&D Systems), in accordance with the manufacturer’s instructions.

### Treatment with the PI3Kγ inhibitor, AS605240

The potential pharmacological role of PI3Kγ blockage in the development of ECM was assessed in infected C57BL/6 mice using ATP-competitive PI3Kγ inhibitor, AS605240 (Sigma-Aldrich, St. Louis, USA) [13, 15–17]. The drug was administered orally (30 mg/kg) and daily from day 3 until day 6 p.i. (a time point when infected WT mice showed neurological symptoms of ECM). AS605240 was suspended in 0.1% methylcellulose solution and control animals received drug vehicle.

### Statistical Analysis

Unless otherwise indicated, data are given as mean ± SEM, indicated by error bars. Differences between infected groups were analyzed by 2-sample T-test (parametric) or Mann Whitney (non-parametric) test when appropriate, using GraphPad Prism 6 (GraphPad Software, La Jolla, California, USA). Survival rates were compared by a log-rank test. p< 0.05 was considered statistically significant.

## Results

### Absence of PI3Kγ confers partial protection against ECM development

**Fig 1:**
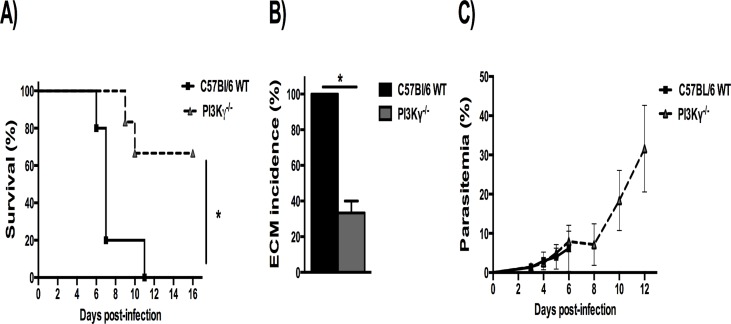
Increased resistance in PI3Kγ^-/-^ mice infected with PbA. Percentage of survival in PbA-infected mice **(A)**. C57BL/6 WT (solid lines, n = 5) and PI3Kγ^-/-^ mice (dashed lines, n = 6). The survival curve of PI3Kγ^-/-^ was significantly different of WT, with *p<0.05. Percentage of ECM incidence in infected mice **(B)** based on typical neurological symptoms of ECM, with *p<0.05 between groups. Parasitemia levels **(C)** from day 3 p.i. up to day 12 p.i., without differences between infected groups. Parasitemia was expressed as mean ± SD. Representative results from 2 independent experiments.

The role of PI3Kγ during PbA infection was assessed by investigating survival, ECM incidence and parasitemia in infected WT and PI3Kγ^-/-^ mice. Infected WT mice developed typical neurological symptoms of ECM including postural disorders, ataxia, loss of grip strength, progressive paralysis, coma, dying on day 6 p.i. (Fig. 1A). In contrast, the majority of infected PI3Kγ^-/-^ mice survived longer than WT mice (p<0.05), without signs of ECM (Fig. 1B). Parasitemia levels were similar between the groups from day 3 p.i. up to day 6 p.i. (Fig. 1C).

### Absence of PI3Kγ attenuates brain histopathological changes

**Fig 2:**
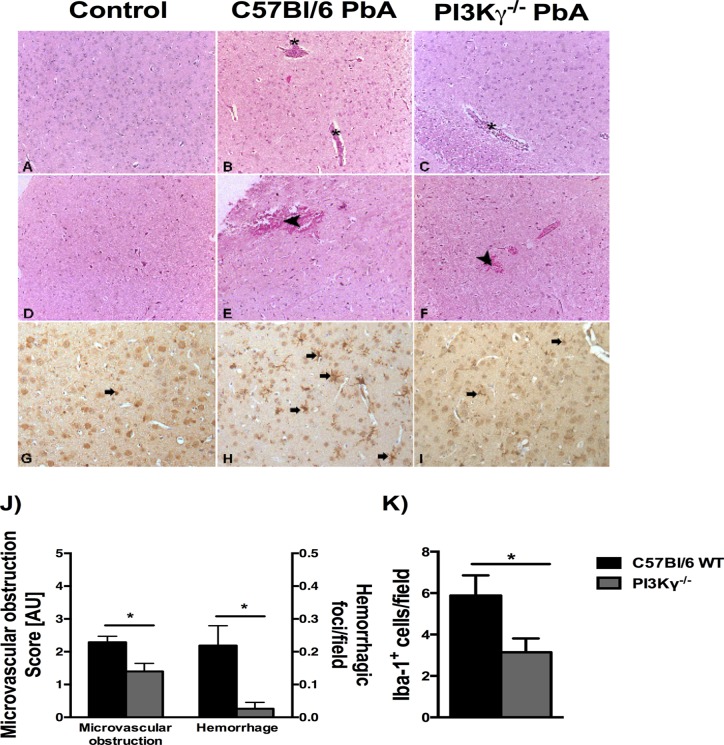
Infected PI3Kγ^-/-^ mice present mild histopathological changes in brain. Representative photomicrographs of hematoxylin/eosin (HE)-stained brain sections from uninfected control mice **(A, D),** or PbA-infected C57BL/6 **(B, E)** and PI3Kγ^-/-^ **(C, F)** mice on day 6 p.i. Normal histological appearance of cerebrum **(A)** and brainstem **(D)** from uninfected control mice. Brain sections from a PbA-infected wild-type animal exhibiting intravascular leukocyte aggregates (asterisks) in cerebrum **(B)** and hemorrhage (arrow head) in brainstem (E). Note vascular plugging (asterisk) in the deep cerebral cortex **(C)** and small hemorrhagic foci (arrow head) in the brainstem **(F)** from PbA-infected PI3Kγ^-/-^ animal. The severity of microvascular obstruction and cerebellar hemorrhagic areas (μm^2^/field) in infected mice demonstrated a significant reduction in brain alterations in PI3Kγ^-/-^ mice **(J)**. Immunohistochemical staining for anti-Iba-1 showing no reactive microglia (arrow) in non-infected WT mouse brain **(G)**. Microglial cells (arrows) displaying morphological changes (thickening of the microglial processes) and immunoreactivity for Iba-1 in the cerebrum of PbA-infected wild-type animal (H) and PI3Kγ^-/-^ mouse (I). Quantification of reactive **microglia (K)** confirmed lower number in brain sections of infected-PI3Kγ^-/-^ mice when compared with PbA-infected WT animal. Significant differences were indicated by *p<0.05 (n = 5 mice per group). Original magnification: A-I: x200. Sections were captured with a digital camera (Optronics DEI-470) connected to a microscope (Olympus IX70).

To determine histopathological changes during ECM, we examined the brains of infected mice on day 6 p.i., when mice presented characteristic neurological symptoms of ECM (Fig. 1A). In infected WT mice, intravascular leukocyte accumulation and perivascular hemorrhage were common findings in the brain (Fig. 2B and E). The severity of brain microvascular obstruction and hemorrhage was assessed semiquantitatively, and infected PI3Kγ^-/-^ mice presented less intense histopathological changes in brain than infected WT mice (Fig. 2C, F and J). These data suggest that absence of PI3Kγ induces less leukocyte accumulation and hemorrhage during PbA infection. During PbA infection, there is microglial activation as demonstrated by immunoreactivity for Iba-1 and cellular morphological changes with thickening of the microglial processes (Fig. 2H). Infected PI3Kγ^-/-^ mice displayed significant less microglia activation when compared with infected WT mice (Fig. 2H, I and K).

### Inactivation of the PI3Kγ pathway reduced cerebral CD8+ T-cell sequestration, CD8+ T-cell cytotoxicity and activation

**Fig 3:**
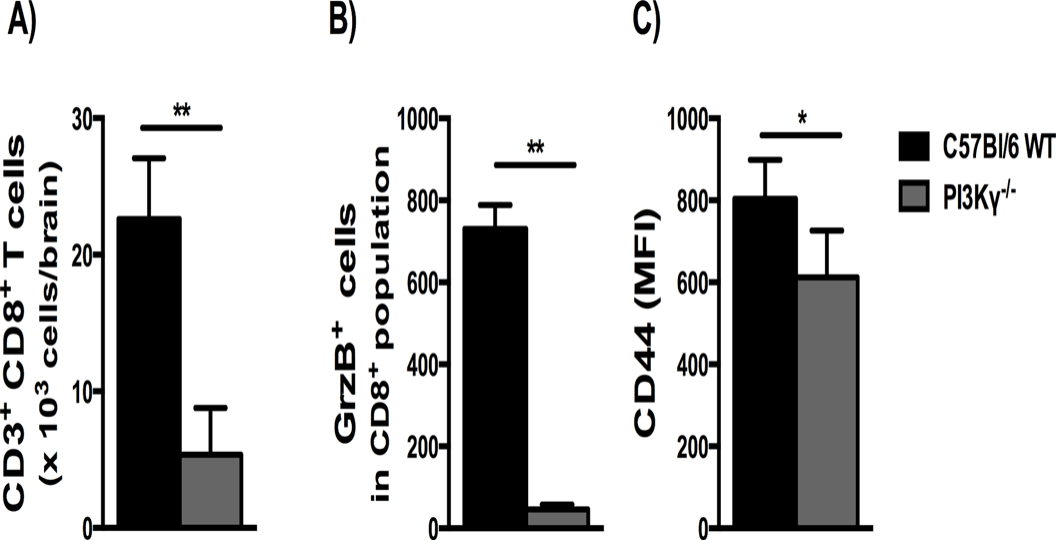
Reduced CD8^+^ T cell accumulation, CD8+ T-cell cytotoxicity and activation in the brain of PbA-infected PI3Kγ^-/-^ mice. Flow cytometric analyses of brain-sequestered leukocytes from PbA-infected WT and PI3Kγ^-/-^ mice on day 6 p.i. Absolute numbers of CD3^+^CD8^+^ T cells, expressed as total number per brain **(A)**. Percentage of Granzyme B^+^ (GrzB^+^) cells in CD8^+^ T population **(B)** and the expression of CD44 in CD8^+^ T cells **(C)** presented as mean fluorescence intensity (MFI). Results are expressed as mean ± SD and are representative of 2 independent experiments (n = 4 samples per group, with each sample representing a pool of 2 mice brains). Significant difference between infected groups was indicated by *p<0.05 or **p<0.01.

The recruitment of T lymphocytes into the brain is necessary for the development of ECM [6, 10], while PI3Kγ is involved in T cell recruitment under certain circumstances [14, 15]. Thus we measured the sequestration of CD3^+^CD8^+^ T cells in the brain as an index of cellular recruitment. Flow cytometry data analysis showed a significant increase of CD3^+^CD8^+^ T cells in the brain of infected WT mice at 6 day p.i., with higher levels of these cells when compared with infected PI3Kγ^-/-^ mice (Fig. 3A). In addition, we measured the expression of Granzyme B (GrzB) and the state of activation of CD8^+^ T cells by the expression of CD44. Infected PI3Kγ^-/-^ mice presented lower percentage of CD8^+^ GrzB^+^ cells and lower CD44 expression (MFI) on CD8^+^ T cells than infected WT mice (Fig. 3B-C).

As IFNγ and CCL5 play an important role on leukocyte migration in the brain during ECM, we also measured the levels of these cytokines in the serum of control and infected mice (S1 A-B Fig.). However, no difference in cytokine levels was observed between infected WT and PI3Kγ^-/-^ mice on day 6 p.i.

### PI3Kγ deficiency attenuates NF-κB activation and Granzyme B levels in brain on PbA infection

**Fig 4:**
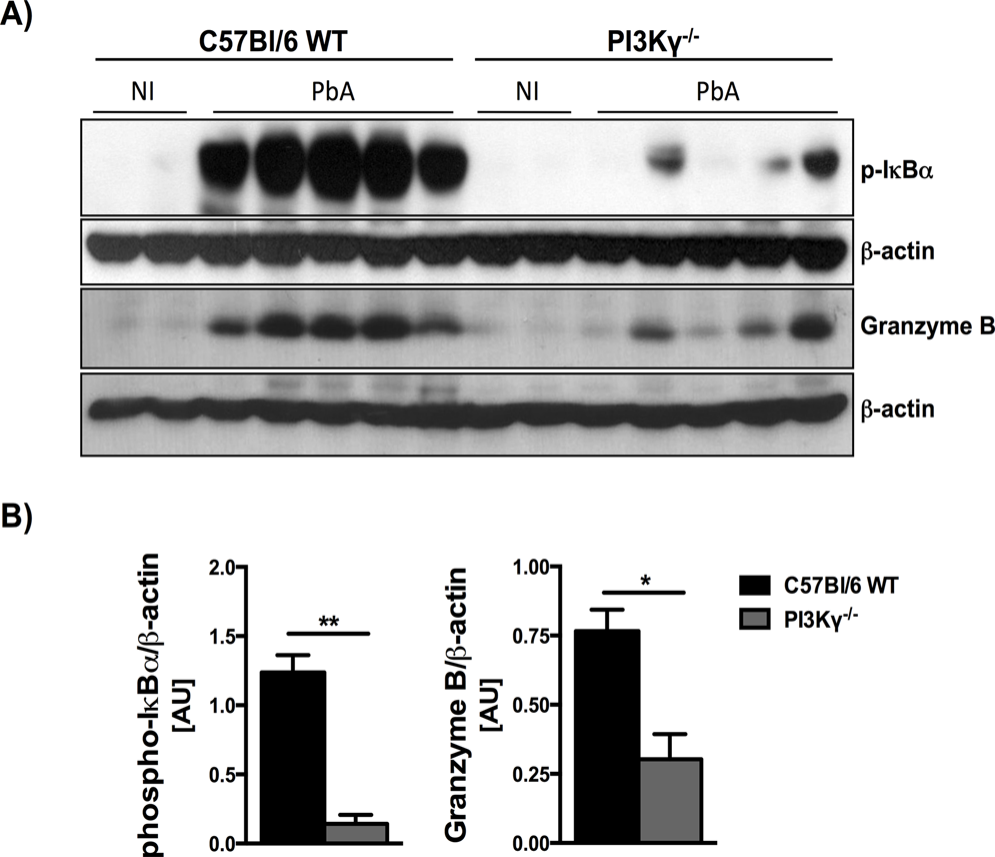
Reduced protein levels of phospho-IκB-α and Granzyme B in brain extracts of infected PI3Kγ^-/-^ mice. Western Blot analysis of phospho-IκB-α (40kDa) and Granzyme B (30kDa) in whole brain extracts, as a marker of NF-κB activation and T cell cytotoxicity, respectively. Samples from WT and PI3Kγ^-/-^ mice (n = 2 for non-infected mice and n = 5 for PbA-infected mice, day 6 p.i.) were collected and processed for protein extraction. Brain extracts (40g) were fractioned on 10% SDS-PAGE, transferred onto nitrocellulose membrane, and then probed with anti-phospho-IκB-α, Granzyme B or anti--actin antibody, for loading control **(A)**. Densitometric analysis are presented as arbitrary units **(B)** and revealed marked increase of phospho-IκB-α and Granzyme B at day 6 p.i., in infected WT mice. The expression of these two proteins was significantly lower in infected PI3Kγ^-/-^ mice when compared with infected WT mice. Results are expressed as mean ± SEM and are representative of 2 independent experiments. Significant differences were indicated by *p<0.05 or **p<0.01.

Using protein extract from the whole brain tissue, we measured the protein levels of Granzyme B and phospho-IκB-α during PbA infection (Fig. 4A) to assess, respectively, T cell cytolytic response and NF-κB activation. In infected WT mice, densitometry measurement revealed marked increase of Granzyme B and phospho-IkB-α at day 6 p.i. In the absence of PI3Kγ, the levels of these two proteins were significantly lower (Fig. 4B).

### Lung pathology is still present in PI3Kγ-deficient mice on PbA infection

**Fig 5:**
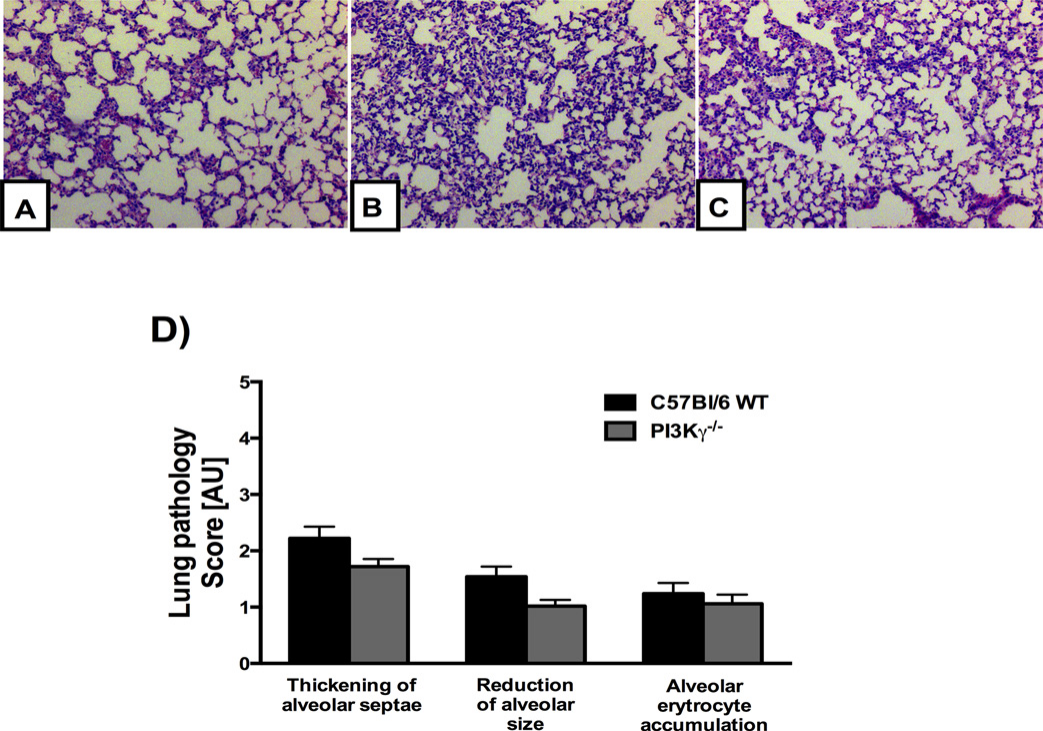
Lung pathology induced by PbA infection seems to be independent of PI3Kγ. Representative photomicrographs of hematoxylin/eosin (HE)-stained lung sections from uninfected control mice **(A),** or PbA-infected C57BL/6 **(B)** and PI3Kγ^-/-^ **(C)** mice on day 6 p.i. Normal histological section of lung parenchyma with standard architecture from uninfected control **(A)**. Lung sections from a PbA-infected C57BL/6 and PI3Kγ^-/-^ animal exhibiting slight interstitial edema, thickening of alveolar septae and a reduction of alveolar size **(B, C)**. Semi-quantification score for histopathological changes in the lung of PbA-infected mice showed no significant difference between C57BL/6 and PI3Kγ^-/-^ infected mice (n = 5 mice for each group) **(D)**. Original magnification: A-D: x200. Sections were captured with a digital camera (Optronics DEI-470) connected to a microscope (Olympus IX70).

During PbA infection, mice also exhibit malaria-associated lung pathology, a process dependent of macrophages and T cells [11, 23, 24]. The lungs of PbA-infected mice displayed congested capillaries of the alveolar septae, hemorrhage in the alveoli and interstitial edema (Fig. 5B and C). We investigated the effects of the PI3Kγ disruption during malaria-associated pulmonary disease and histopathological analysis found no significant difference between infected WT and infected PI3Kγ^-/-^ mice. Therefore, lung pathology induced by PbA infection seems to be, at least partially, independent of PI3Kγ.

### Treatment with an inhibitor of PI3Kγ delayed mortality in WT PbA-infected mice

**Fig 6:**
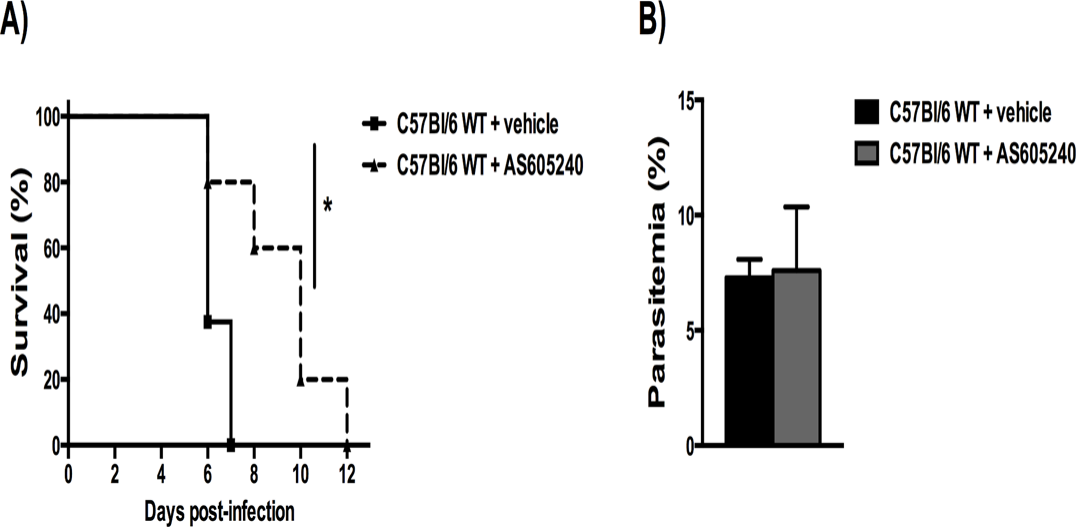
Treatment with a PI3Kγ inhibitor delayed mortality in WT PbA-infected mice. The selective inhibitor of PI3Kγ, AS605240, was given orally, once a day, at the dose of 30 mg/kg, from day 3 until day 6 p.i. (n = 5). Untreated infected animals (n = 8) received drug vehicle in the same therapeutic scheme. Percentage of survival in PbA-infected mice **(A)**, where solid lines denote vehicle-treated mice and dashed lines, AS605240-treated mice. Parasitemia levels on day 6 p.i., without differences between infected groups **(B)**. Significant differences were indicated by *p<0.05 and parasitemia expressed as mean ± SD.

Because lack of PI3Kγ protected partially against ECM, next we investigated whether similar protection could be attained with a pharmacological strategy given after the infection. The potential role of PI3Kγ blockade in the development of ECM was assessed in infected C57BL/6 mice using a selective inhibitor of PI3Kγ, AS605240, given daily from day 3 until day 6 p.i. As seen in Fig. 6, treatment with the PI3Kγ inhibitor significantly delayed lethality (Fig. 6A). Akin to results seen in PI3Kγ^-/-^ mice, parasitemia levels on day 6 p.i. were similar between treated and non-treated infected mice (Fig. 6B).

## Discussion

The current study provides the first evidence that mice lacking PI3Kγ are more resistant to ECM induced by PbA infection. PI3Kγ-deficient mice exhibited mild clinical sings and lower inflammatory changes in the brain (i.e., microglia activation, T cell cytotoxicity and CD8^+^ T cells sequestration), but no changes in parasitemia levels or lung pathology. The pharmacological blockade of PI3Kγ significantly delayed mortality of animals subjected to ECM, even when drug treatment was started 3 days after infection, confirming the relevant role of PI3Kγ in ECM pathogenesis and the potential of blocking this pathway to prevent and/or delay severe disease.

PI3Ks mediate intracellular signaling and are involved in several cellular processes, including the recruitment and activation of T cells. PI3Kγ plays a pivotal role in the motility of leukocytes in several models of inflammation. Inhibition of PI3Kγ has been considered a potential therapeutic strategy in several animal disease models [13, 15–17]. For instance, using PI3Kγ-deficient mice, Rodrigues et al. [25] found a reduction of clinical symptoms in experimental autoimmune encephalomyelitis (EAE), associated with decreased inflammatory infiltrate in the brain. Recently, Li et al. [26] demonstrated that PI3Kγ deletion and the systemic treatment with another selective PI3Kγ inhibitor (AS-604850) significantly reduced brain inflammation and ameliorated the clinical symptoms of EAE mice. In addition, PI3Kγ deficiency significantly reduced blood-brain barrier permeability and brain edema formation in a model of experimental stroke [27].

The development of ECM pathogenesis after PbA infection is dependent on CD8^+^ T cell-mediated cytotoxic mechanisms [7, 9, 10]. Interestingly, a recent work showed that PbA infection induces brain microvessels to cross-present parasite antigen to CD8^+^ T cells, while non-ECM-causing parasites do not [28]. Several reports have documented microglia activation in the brain of PbA-infected mice [29, 30]. In line with these studies, our work revealed that the absence of PI3Kγ partially protected mice against ECM with less intense brain pathology, reduced microglia activation, lower levels of Granzyme B, and less CD8+ T-cell sequestration/accumulation into the brain. Leukocyte recruitment and activation may be dependent on PI3Kγ and we observed lower expression of CD44 in CD8+ T cell population and lower levels of phospho-IkB-α in the whole brains of infected PI3Kγ^-/-^ mice when compared with infected WT mice. Using a bleomycin mice model for idiopathic pulmonary fibrosis, Russo et al. [31] showed attenuated AKT phosphorylation and NF-κB activation in PI3Kγ-deficient mice which were associated with reduced pulmonary fibrosis and lower lethality in these mice. Therefore, prevention of lymphocyte sequestration, associated with lower CD8+ T cell cytotoxicity and microglial activation may underlie the mechanisms by which the absence of PI3Kγ protects mice from severe malaria.

Previously, our group has demonstrated that Platelet-Activating Factor Receptor (PAFR) appears to contribute to both pulmonary and cerebral inflammation in PbA-infected mice [20, 32]. However, resistance to ECM is not associated with resistance to malaria-associated lung pathology [11]. Fauconnier et al. [19] showed that accumulation of pathogenic CD8+ T cells in the brain vasculature after PbA infection is PKC-θ-dependent, but PKC-θ deficiency does not protect against PbA-induced lung disease. In the current study, the absence of PI3Kγ was not able to prevent lung pathology, although prevented T cell sequestration during cerebral inflammation. Since malaria-associated lung pathology is mediated by T cells and other cell types, mainly macrophages [11], the involvement of these other cells during PbA-induced lung disease could explain the absence of pulmonary protection in PI3Kγ-deficient mice. In addition, liver damage was subtle in PbA infected mice at day 6 p.i. (data not shown), with no significant difference between infected WT and PI3Kγ^-/-^ infected-mice. Taken together, PI3Kγ signaling is important for the development of ECM but it does not seem essential for other systemic manifestations of PbA infection. IFN-γ and chemokines play a major role in driving leukocytes to the brain after PbA infection, however no difference between infected mice was observed for IFN-γ and CCL5 serum levels.

Our results clearly indicate that PI3Kγ signaling is required for the development of ECM induced by PbA infection. To demonstrate whether ECM is amenable to drug intervention by drugs that affect PI3Kγ, we performed experiments using AS605240, a selective PI3Kγ inhibitor. Previous studies demonstrated a significant protective effect of AS605240 in different mouse models, such as systemic lupus erythematosus, graft-versus-host disease, autoimmune diabetes and rheumatoid arthritis [13, 15–17]. In the current work, even when started 3 days after infection, AS605240 treatment delayed lethality in WT mice after PbA infection. Of note, the drug did not alter parasitemia, as also observed in PI3Kγ-deficient mice. Further studies using different therapeutic regimens (e.g., increasing the time of treatment and/or starting before or after day 3 p.i.) are warranted. In conclusion, we suggest that strategies aimed at blocking the action of PI3Kγ could be useful as an adjuvant therapy in patients with malaria infection.

## Supporting Information

S1 Fig Levels of IFNγ and CCL5 in the serum were similar in WT and PI3Kγ^-/-^-infected mice. Analysis of IFNγ **(A)** and CCL5 **(B)** levels in the serum of WT and PI3Kγ^-/-^ mice. Similar levels between WT and PI3Kγ^-/-^-infected mice on day 6 p.i. Results are expressed as mean ± SEM.
(TIFF)
