# Supplementary figures and images for: Phosphatidylinositol 3-Kinase γ Is Required for the Development of Experimental Cerebral Malaria
Source: PLoS One. 2015 Mar 16;10(3):e0119633. doi: 10.1371/journal.pone.0119633 (PMC4361544; doi:10.1371/journal.pone.0119633)

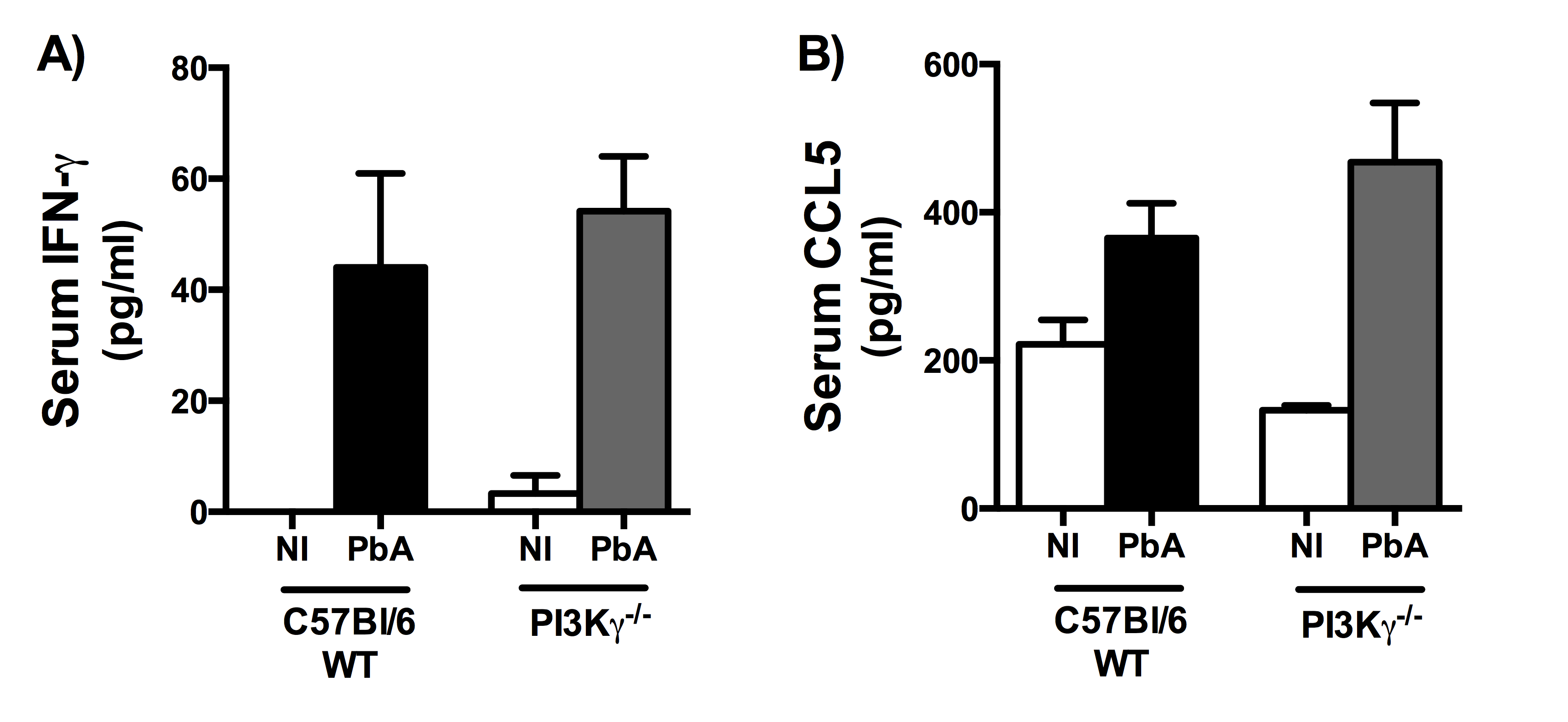

Supplement: S1 Fig — Analysis of IFNγ (A) and CCL5 (B) levels in the serum of WT and PI3Kγ-/- mice. Similar levels between WT and PI3Kγ-/--infected mice on day 6 p.i. Results are expressed as mean ± SEM. (TIFF) [file pone.0119633.s001.tiff]
